# Quantifying the effect of environment stability on the transcription factor repertoire of marine microbes

**DOI:** 10.1186/2042-5783-1-9

**Published:** 2011-09-07

**Authors:** Ivaylo Kostadinov, Renzo Kottmann, Alban Ramette, Jost Waldmann, Pier Luigi Buttigieg, Frank Oliver Glöckner

**Affiliations:** 1 Max Planck Institute for Marine Microbiology, Celsiusstrasse 1, 28359 Bremen, Germany; 2 Jacobs University Bremen gGmbH, Campus Ring 1, 28759 Bremen, Germany

**Keywords:** transcription factors, ecological metagenomics, interpolated environmental data, multivariate statistics

## Abstract

**Background:**

DNA-binding transcription factors (TFs) regulate cellular functions in prokaryotes, often in response to environmental stimuli. Thus, the environment exerts constant selective pressure on the TF gene content of microbial communities. Recently a study on marine *Synechococcus *strains detected differences in their genomic TF content related to environmental adaptation, but so far the effect of environmental parameters on the content of TFs in bacterial communities has not been systematically investigated.

**Results:**

We quantified the effect of environment stability on the transcription factor repertoire of marine pelagic microbes from the Global Ocean Sampling (GOS) metagenome using interpolated physico-chemical parameters and multivariate statistics. Thirty-five percent of the difference in relative TF abundances between samples could be explained by environment stability. Six percent was attributable to spatial distance but none to a combination of both spatial distance and stability. Some individual TFs showed a stronger relationship to environment stability and space than the total TF pool.

**Conclusions:**

Environmental stability appears to have a clearly detectable effect on TF gene content in bacterioplanktonic communities described by the GOS metagenome. Interpolated environmental parameters were shown to compare well to *in situ *measurements and were essential for quantifying the effect of the environment on the TF content. It is demonstrated that comprehensive and well-structured contextual data will strongly enhance our ability to interpret the functional potential of microbes from metagenomic data.

## Background

Microorganisms constantly adapt to their environment to survive. An efficient response mechanism is the regulation of transcription, the first step in gene expression, according to environmental demands. Transcription factors (TFs) are the primary agents that perform transcriptional regulation [1]. They consist of a DNA-binding domain (DBD) that typically targets regulatory elements upstream of a gene and an effector domain [2]. The majority of TFs operate by influencing the downstream transcription process and can be classified into 10 super-families according to their DNA-binding mechanisms [3]. Based on the number of genes they regulate, TFs can be divided into 'global regulators' and 'fine tuners' [4]. Both types exert targeted control over gene expression. Global regulators affect a larger number of genes from diverse metabolic pathways and respond to a wider set of stimuli [4,5]. Conversely, fine tuners are triggered by more specific stimuli and control fewer genes. Up to 10% of bacterial gene products may be devoted to gene regulation [6], a proportion supported by *in silico *analysis of TF abundance in 123 bacterial and archaeal genomes [7]. Although the maximum number of TFs in prokaryotic genomes is bound by the degrees of freedom in their binding mechanisms, larger genomes tend to have more TFs [1,3]. A greater number of TFs may enable more precise control of gene expression which is required by a complex lifestyle [6]. In general, free-living *Bacteria *and *Archaea *from dynamic environments possess more TFs than those from stable environments [8]. Recently, the effect of environmental factors on gene expression has been studied in the marine model organism *Rhodopirellula baltica *SH1^T ^[9]. Although only 2% of its gene content is dedicated to transcriptional control [10], it showed a fine-tuned regulation response to environmental stress.

Palenik and co-workers (2006) reported that the gene content of two marine *Synechococcus *strains, one isolated from coastal waters and the other from the open ocean, reflect the variability of their respective environments [11]. The coastal strain possessed a higher number of sensors and response regulators when compared to the open ocean strain, allowing it to respond to its dynamic environment. Gianoulis and coworkers (2009) investigated the environmental adaptation of metabolic pathways in the Global Ocean Sampling (GOS) metagenomes [12]. They observed no significant differences in the abundance of transcriptional/translational pathways between these two groups of samples, loosely described as open ocean and coastal. A more recent study described environmental adaptation in 197 marine microbial genomes and related the findings to the GOS metagenome [13]. The abundant cosmopolitan species which are adapted to slow growth in nutrient-poor conditions have a smaller genome size, lower metabolic plasticity, and fewer transcriptional regulators than their counterparts which are adapted to alternating periods of 'feast and famine'. However, quantifying the effect of the environment on the transcription factor repertoire of marine microbes remains a challenge. A comprehensive set of environmental parameters, describing the samples at the time they were taken and the sampling location over monthly to yearly time scales, is a prerequisite for addressing this question. Unfortunately, environmental *in situ *measurements taken during sampling are often missing or incomplete. Even when they are at hand, they give only a static 'snapshot' of the environmental conditions. The use of interpolated parameters can help to overcome these shortcomings: they can replace missing values, describe sampling sites in different temporal scales and give indications of the stability of the environment. A few metagenomic studies have taken advantage of these features of interpolated environmental parameters. Gianoulis and coworkers (2009) validated imputed salinity values against extrapolations from the World Ocean Database [14]. Rusch and coworkers (2010) used monthly averages for nitrate and phosphate from the World Ocean Atlas (WOA) to study the *Prochlorococcus *clades detected in the GOS metagenome with respect to nutrient availability [15].

Here we investigated the influence of environment stability on the relative number of different TFs (TF content) in samples from the GOS metagenome [16,17]. To this end, we (1) compared interpolated environmental parameters against on-site measurements to verify the predictive power of the interpolations used; (2) calculated a yearly stability measure for each environmental parameter based on 12 monthly averages; (3) applied redundancy analysis (RDA) to assess the effect of environmental stability and spatial distance (i.e. space) on the TF content; (4) used multiple linear regression (MLR) to identify possible dependencies between single TFs, combinations of stability parameters, and space.

## Results and Discussion

### Interpolated environment parameters compare well to *in situ *measurements

**Figure 1:**
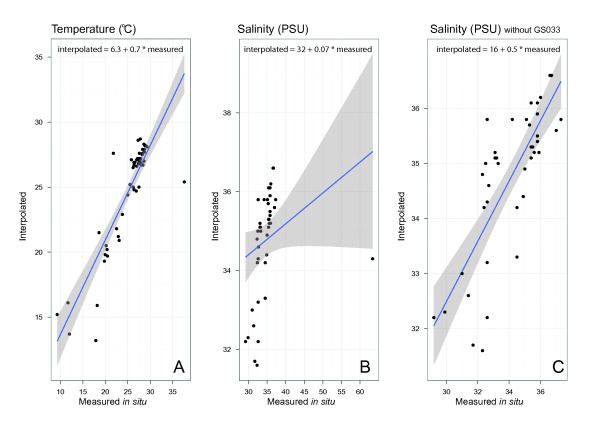
**Linear regression analysis of measured and interpolated environmental parameters**. Temperature (A), salinity (B) and salinity with sample GS033 removed (C). The points represent the samples. The solid blue line is the fitted linear function and the shaded area depicts the confidence interval for it.

We selected GOS samples where on-site measurements and monthly interpolated values for temperature (55 samples) and salinity (44 samples) were available. We used a linear regression model using interpolated monthly parameter values to predict values measured on board the Sorcerer II during sampling. Both interpolated temperature and salinity values proved to be good estimators of the measured values, with a goodness-of-fit value (R^2^) of 0.76 (p-value < 2.2e-16) and 0.6 respectively (p-value = 2.459e-10) (Figure 1). Coastal areas, however, pose a significant problem for interpolation due to lack of reliable data or major terrestrial influences on the water bodies that are hard to quantify (e.g. riverine input, anthropogenic activity). Sample GS033 came from a hypersaline mangrove forest, an environment that differs markedly from the surrounding water masses. The interpolated monthly average for this sample was 29 Practical Salinity Units (PSU) lower than the measured one. Considering that the area is known to be hypersaline, this large difference is more likely due to an insufficient number of data points available for interpolation rather than by a temporary event taking place at the time of sampling. Supporting this assumption, the interpolated monthly temperature was 12°C lower than the *in situ *measurement. Because no reliable interpolations were possible for GS033, it was excluded from the regression analysis of salinity and from the environment stability analysis. The combination of numerical data with categorical description (hypersaline) of the habitat helped to detect and explain differences between interpolated and *in situ *values. The interpolations for the remaining locations are based on a number of previous *in situ *measurements [18] and easily accessible surface waters, i.e. the first 30 m of the marine epipelagic zone, are well sampled in this regard. This is the probable reason for the good fit between measured and interpolated monthly values. Our results suggest that numeric interpolation of environmental parameters can complement or, when necessary, even substitute parameters measured *in situ*. These comprehensive datasets can then be used, with a fair degree of confidence, in deriving more complex descriptors of the environment such as its stability.

### Variation in single-copy gene numbers

Single-copy genes (SCGs) are genes which are assumed to appear only once per genome. Their total number is suggested to reflect the genome equivalents in metagenomic samples [19]. Therefore, they are good candidates to standardize results of sequence-based searches in samples of different sizes. However, we expected significant differences in the occurrences of different SCGs because of sequencing bias. To test this assumption, we compared the abundance of 53 prokaryotic SCGs in 58 GOS samples. Four overrepresented and 12 underrepresented SCGs were found (Additional file 1, Figure S1 and Additional file 1, Table S1). Some of those were outliers in up to 98% of the samples. Over- and under-representation of SCGs was observed in all samples, although the variation dropped with increasing number of sequences per sample (Additional file 1, Figure S1 and Additional file 1, Figure S2).

We compared the behavior of basic statistical descriptors like the mean and the median for producing a suitable standardization parameter (Additional file 1, Figure S3). All descriptors behaved in a similar way, showing an increasing number of SCGs with increasing number of sequences. The interquartile range remained stable regardless of the sample size, showing an almost equal spread of the SCG counts per sample. We performed the analysis of the total TF content using two standardization parameters corresponding to two standard deviations above and below the mean and compared the results. No significant difference was detected, and even if such a difference was observed, using both parameters for calculations would translate into reporting results as a range rather than as a single value.

It is possible that cloning and sequencing biases in the GOS metagenome may explain over- and underrepresentation of certain SCGs. It is also possible that some of the SCGs appear in more than one copy in some genomes. The original work of [20] that identified SCGs was based on 191 completely annotated genomes across the tree of life. At the time of our study, the ENTREZ Genome Project collection http://www.ncbi.nlm.nih.gov/genomes/lproks.cgi listed 1446 complete microbial genomes and another 3888 in progress. Furthermore, an EnvO-Lite [21] classification of complete microbial genomes available at the megx.net portal http://www.megx.net features 227 marine water column isolates. Given the many-fold increase in microbial genomes, it would be beneficial to re-evaluate the list of SCGs, focusing on marine prokaryotes, but such analysis was beyond the scope of this study. According to [22], the average genome size of a sample and the length of an SCG influence relative counts. The SCGs used here are universally distributed, most of them being related to the translation machinery [20]. Therefore, their presence should be genome-size independent. The effect of gene length on the sampling probability is neutralized by combining the observations from several SCGs with different lengths. Ultimately, we used the mean SCG count per sample as a standardization measure.

### The TF content significantly responds to environment stability

We derived eight environment stability measures based on the standard deviation of interpolated monthly temperature, salinity, dissolved oxygen, apparent oxygen utilization (AOU), oxygen saturation, phosphate, nitrate, and silicate measurements over a 12-month period. This was done for 44 of the samples used for the determination of the SCG variation. Because co-varying stability measures may confound statistical analyses, we only retained variables with a correlation coefficient below 0.6 to any other variables (Additional file 1, Table S2). As expected, nitrate stability correlated strongly with phosphate stability. The tight connection between these two nutrients is well known as the Redfield Ratio [23]. Tyrell (1999) showed the strong correlation between phosphate and nitrogen in the WOA data [24]. Similarly, the amount of dissolved oxygen is known to depend strongly on water temperature [25]. This relationship showed as a strong correlation (ρ = 0.75) between the two stability measures. Oxygen saturation and AOU are both derived from the dissolved oxygen [26] but they showed exceptionally high correlation (ρ = 0.99) to each other and moderate correlations to either phosphate (ρ = 0.63) or silicate (ρ = 0.61). Thus, the stability measures for temperature, salinity, phosphate, and silicate were used for further analysis.

**Figure 2:**
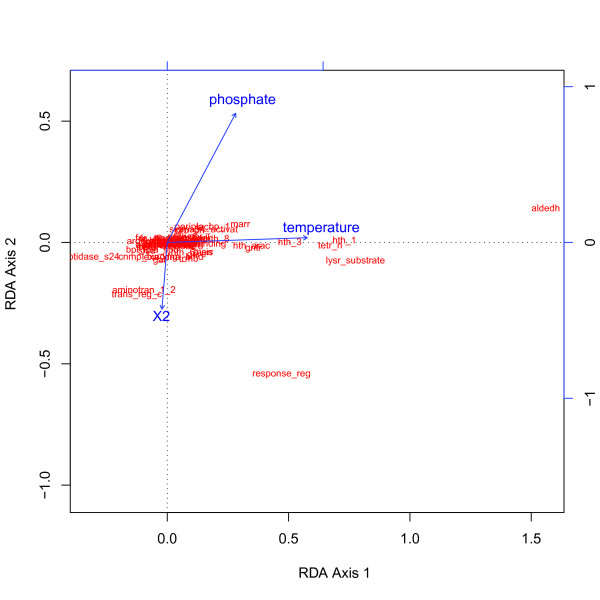
**RDA biplot of TFs constrained by environment stability and space**. The ordination of TFs (in red) constrained by the explanatory variables (blue vectors) is shown. The lengths of the vectors correspond to the strength of the effect of that particular variable. RDA scaling 2 was used (scaling the TF scores). The angle between an explanatory variable vector and a TF (if a vector was to be drawn from the origin of the graph to this TF) approximates their correlation.

In order to evaluate the effect of the environment stability on the total TF content in 44 GOS samples we used RDA. Combining automatic and manual parameter selection, we found a statistical model in which environment stability and space best described the differences in TF content between the samples (TF variation). The environment stability was represented by temperature stability (p-value < 0.001) and phosphate stability (p-value < 0.1) and accounted for 35% of the variation in TFs. Of that, 28% were contributed by temperature stability (p-value < 0.001), 2% by phosphate stability (p-value < 0.05), and 5% by a combination of both. The contribution of phosphate stability is moderate compared to temperature, yet statistically significant and should be taken into consideration. As described above, for pairs of strongly correlating stability measures only one measure was taken; therefore, the effects of two strongly correlating parameters could not be differentiated. Temperature stability could either influence TF variation directly or indicate another influencing factor correlating with temperature. For example the correlation dissolved oxygen with temperature is well known and has an ecological significance. The same is true for phosphate stability and nitrate stability. Tyrrell (1999) argues that phosphate limits oceanic primary production on a short time scale, while nitrate limits it on a global time scale [24]. In this study, we cannot speculate on what time scale environmental changes cause genomic TF variation in prokaryotes. Spatial distance was represented by one of the two axes (X2), produced by principal coordinate analysis of the Cartesian distances between samples and accounted for 6% of the TF variation (p-value < 0.01). Because many TFs perform universal house-keeping functions, spatial distance alone was expected to explain only a minor proportion of the TF variation. In this case, space could be considered an abstract proxy for the different conditions between spatially separated environments. Testing the effect of space separately ensures that the effect of environmental stability is not influenced by other factors that differ between samples purely due to spatial distance. Contrary to our expectations, no variation could be explained by the combined effect of environment stability and space in our model. A biplot of the RDA results reveals that the majority of TFs cluster together and the explanatory variables do not have enough discriminatory power (Figure 2). However, several TFs like a family of dehydrogenases acting on aldehyde substrates (Aldedh, PF00171) were more strongly affected by the environment stability and space. Overall, 59% of the variation in the TF content remained unexplained and it is clear that further factors are required to explain patterns of TF distribution more completely. Additional environmental parameters, taxonomic composition and interactions with viruses and eukaryotes are likely to feature among these.

Gianoulis and coworkers (2009) explored the adaptation of metabolic pathways in the GOS metagenome to the environment [12]. They divided the samples in two groups, loosely described as coastal and open ocean. No significant difference in the transcription machinery between two sets was detected. In their estimation, fine-grained relationships between the samples and their environment might have been undetectable by the method used to partition the samples. Although generally similar, our study differs from that of Gianoulis et al. (2009) in several aspects. Their explorative approach was well suited for a broad range of pathways. However, more subtle patterns in specific pathways might remain undetected. Here we focused on one functional group (TFs) and adapted our methods accordingly. We performed the analysis on a six-frame translation of the raw GOS reads to avoid artifacts from assembly and ORF prediction. Further, we used a curated list of Hidden Markov Models (HMM) to detect genes of interest and used an extended set of environmental parameters, including nutrients. Small-scale differences along nutrient gradients are of importance when describing the ecology of microorganisms [27], so we kept the scale as fine-grained as possible. Lastly, we investigated the adaptation of microbial TF repertoire in response to environment stability rather than temporary environmental conditions. We were able to complement the findings of Gianoulis et al. (2009) with a detailed quantification of the TF content adaptation to environmental stability.

A more recent study detected the trend in the TF repertoire of marine microbes we quantified here [13]. The genomes of 197 marine isolates were compared with respect to their coverage in the GOS dataset resulting that only 34 marine genomes are well covered in the GOS dataset. These are very streamlined, having heavily reduced capacities for transcriptional regulation, environment sensing and amino-acid uptake. The remaining 163 genomes were sparsely covered by the GOS dataset and were more adapted to changing environmental conditions. Yooseph and coworkers concluded that the prevailing picoplankton has a low 'bacterial IQ' [28] and uses alternatives to transcriptional control for metabolic regulation. Our findings from directly querying the metagenome concur with the differences based on trophic strategies observed by Yooseph et al. (2010). With 35% effect of environmental stability on the TF content we have shown that more dynamic environments require different TF repertoires than stable environments.

### Single TFs are more tightly connected to environment stability and space

The RDA of total TF content suggested that individual TFs show stronger relationships to environment stability than the total TF content. Using the 44 samples we applied MLR to test the effect of environment stability and space on single TFs. For 19 TFs more than 30% of the variation could be explained by a combination of environmental stability parameters and spatial components (Table 1). Temperature stability was present in all MLR models. Temperature is known to be an important factor in determining bacterial populations and their functions in the oceans [29]. However, temperature might also be a proxy for other parameters. Several TFs were best explained (most explained variation) by different combinations of temperature stability, salinity stability and the second spatial axis (X2). Since these factors are rather broad, we inspected more closely the TFs which were co-explained by phosphate (i.e. nutrients) stability and silicate stability.

**Table 1:** Multiple Regression results for single TFs.

TF (non-DBD)	Multiple regression model	R-squared	p-value
response_reg	temperature (p < 0.05) + phosphate (p < 0.01) + X2^2 (p < 0.05)	0.31	1.93E-03

peptidase_s24	temperature (p < 0.001)	0.34	1.67E-02

pro_dh	temperature (p < 0.001) + X1 (p < 0.01)	0.38	6.23E-02

Aldedh	temperature (p < 0.001) + X2 (p < 0.1)	0.46	3.78E-03

Sugar.bind	temperature (p < 0.001) + salinity (p < 0.01)	0.47	2.30E-03

Utra	temperature (p < 0.001)	0.49	1.06E-04

Tobe	temperature (p < 0.001) + salinity (p < 0.05) + silicate (p < 0.05) + X2 (p < 0.05)	0.56	1.22E-03

lysr_substrate	temperature (p < 0.001) + X2 (p < 0.001)	0.58	1.63E-05

**TF (DBD)**	**Multiple regression model**	**R-squared**	**P-value**

Laci	temperature (p < 0.001) + silicate (p < 0.05)	0.30	6.44E-04

Laci	temperature (p < 0.001) + phosphate (p < 0.05)	0.31	4.88E-04

Gntr	temperature (p < 0.001) + X2 (p < 0.05)	0.38	5.61E-02

penicillinase_r	temperature (p < 0.05) + salinity (p < 0.01) + phosphate (p < 0.01) + X2 (p < 0.05)	0.41	3.36E-04

hth_arac	temperature (p < 0.001) + X2 (p < 0.01)	0.41	1.87E-02

hth_6	temperature (p < 0.001) + phosphate (p < 0.05)	0.43	1.03E-05

hth_3	temperature (p < 0.001) + silicate (p < 0.1) + X2 (p < 0.01)	0.52	1.42E-03

tetr_n	temperature (p < 0.001) + X2 (p < 0.01)	0.54	1.10E-04

trp_repressor	temperature (p < 0.001) + phosphate (p < 0.1)	0.55	8.52E-08

Lyttr	temperature (p < 0.01) + silicate (p < 0.05) + X2 (p < 0.01)	0.57	2.22E-04

hth_1	temperature (p < 0.001) + salinity (p < 0.05) + X2 (p < 0.01)	0.60	5.65E-08

Only results with a goodness-of-fit value (multiple R-squared) above 0.3 (30% explained variation) are shown. The significance of each term in the linear model (p-value) is given next to it.

Nutrient stability co-explained the variability of both broad and specific TFs. Response_reg (PF00072) is a general receptor domain which interacts with a DNA-binding effector domain (often LytTR). The model representing LacI (PF00356) family of regulators is a broad-spectrum DBD. This particular TF was equally well explained by temperature stability and either phosphate or silicate stability. We speculate that this is due to the wide range of regulators belonging to this family. Penicillinase_R (PF03965) is responsible for the repression of the penicillinase gene. Availability of nutrients generally causes increased prokaryotic and eukaryotic cell density in the water column. The release of beta-lactam antibiotics is a competitive measure in such a scenario which must be met with a well-regulated resistance. In coastal areas, terrestrial input of such antibiotic substances can also be expected. The HTH_6 domain (PF01418) is involved in the regulation of phospho-sugar metabolism, we speculate that we observed a direct link between the function regulated by the TF and phosphate stability. Production of phosphosugar molecules requires inorganic phosphate. Increased concentrations of inorganic phosphate can be one factor supporting the increase of microbial populations and thus, the production of phosphate-containing compounds like phosphosugars. In dynamic environments with changing nutrient concentrations, phosphosugar molecules will become only temporarily available. Regulating their assimilation based on availability would optimize the energy use of the microbial population.

Another TF, Trp_repressor (PF01371), regulates the Tryptophan operon and is a classic example for transcription control by attenuation. Tryptophan is costly to produce in terms of energy [30]. Microorganisms would profit from switching off the production of tryptophan whenever it is available for uptake from the environment. Tight regulation of tryptophan biosynthesis would be beneficial in environments with dynamic nutrient concentrations, but not in environments with constantly low nutrient concentrations, where it has to be continuously produced.

Silicate stability co-explained the variation in TFs which describe a scenario where bacterial populations interact with eukaryotes in a dynamic environment. TFs from the HTH_3 family (PF01381) are involved in plasmid copy control and methylation, the latter a means to prevent the digestion of DNA by restriction endonucleases mechanism. TOBE (PF03459) is part of ABC transporters and detection of small ligands like sulphate. LytTR (PF04397) is involved in the control of cell autolysis. Bacterial adaptation includes complex interactions with phytoplankton. Bacterial assemblages mediate silicon regeneration from lysed diatoms, detritus and marine snow [27,31]. Algal blooms, for example, strongly affect microbial communities [32,33]. In a bloom situation, precise control over substance detection and transport, defense mechanisms and cell death would provide a selective advantage. Based on the TFs whose variation was co-explained by silicate, we speculate that we have detected a response of bacterial regulatory potential to oscillations in diatom communities, for example during and after an algal bloom.

Our findings on single TFs are in line with the trophic description of the GOS dataset [13]. Typically, copiotrophs are adapted to capitalize on transient nutrient availability on which the survival of their populations strongly depends. They are more influenced by marine eukaryotes (e.g. algal blooms) and dominate the water column only sporadically [13,34]. In contrast to microbes with oligotrophic adaptations, copiotrophs still possess the majority of energy uptake systems (e.g. amino acids). We have shown that variation in nutrients has a significant effect on the number of TFs related to these functions. The environmental stability effect on the three TFs discussed above strongly suggests that these TFs are essential to copiotrophic communities for adapting to their changing environments.

### Detection limits and interpretation considerations with our approach

The Pfam HMMs [35] used in this study model only key protein domains of the TFs and sometimes represent whole TF families. Therefore, an absolute, one-to-one relationship between a single TF and a particular gene or function is sometimes impossible to infer. Although we used a set of eight environmental parameters, other factors (e.g. predator-prey interactions, viral infections, iron concentration) might significantly contribute to the patterns of TF distribution. Moreover, the interpolated environmental data values were monthly averages which might not reflect smaller temporal variations. These constraints form a certain resolution limit on our findings that is hard to quantify. On the other hand, the selective pressure which the environment stability exerts on bacterial transcription control was strong enough to leave a genomic imprint which is detectable despite this resolution limit. Furthermore, metagenomics provides a glimpse into the genomic potential of microbial communities, but not into their gene expression patterns. Therefore, any dependencies between the environment and the genomic repertoire have to be rather stable. In this study, we focused on linear relationships between TF content and the numeric stability of the environment, but non-linear relationships could also be possible.

## Conclusion

Using interpolated environmental data, we detected and quantified an ecogenomic trend in the transcription factor repertoire of marine bacterial communities that depended on spatial distance and environmental stability. Environment stability was responsible for 35% of the variation in total TF content while 6% was attributed to space. Up to 60% of the variation in single TFs could be attributed to combinations of environment stability factors and space. In several cases the function controlled by the TFs was directly related to the environmental stability measures that best explained their variation. Despite resolution limitations of the data, our results strongly suggest that the effect of environment stability on the genome composition of bacterioplankton is a strong, detectable signal. Improved availability and integration of contextual data, preferably compliant with the checklists of the Genomics Standards Consortium [36,37], will make it possible to describe ecogenomic trends with higher resolution and better characterize the influence of the environment on prokaryotic metagenomes.

## Methods

### Sequence and Environmental Data

Sequence reads and metadata for 82 samples of GOS metagenome were obtained from the Community Cyberinfrastructure for Advanced Microbial Ecology Research & Analysis (CAMERA) website [38]. These include samples from the Sargasso Sea [16], the northwest Atlantic, the eastern tropical Pacific [17], and the Indian Ocean transect. The interpolated environmental data for the GOS samples (Additional file 2, Table S1 and Additional file 2, Table S2) was extracted from the portal for Marine Ecological Genomics [39] using the geographic location (based on GPS coordinates), sampling date and depth. The interpolations were based on data from the World Ocean Atlas 2005 [14]. Eight environmental parameters were available, namely temperature, salinity, dissolved oxygen, apparent oxygen utilization (AOU), oxygen saturation, phosphate, nitrate, and silicate. For each environmental parameter a single value is available per degree latitude and longitude on 33 standard depth levels. Inverse distance weighted interpolation were performed based on the GPS coordinates and depth of the samples, originally reported by GOS.

### Ecological modeling

Statistical analyses and plotting were performed using the free software environment for statistical computing and graphics, R [40] with the *vegan *[41], and *MASS *packages [42]. The R code for this study is available in Additional file 2 (Rcode.txt).

For linear regressions of environmental data, all GOS samples where interpolation for temperature and salinity was possible were considered (Additional file 2, Table S1). Only one *in situ *measurement and one interpolated value per sampling site, defined by unique GPS coordinates, time and depth of sampling, are possible. Therefore, only one sample per sampling site was kept. Two samples GS000a and GS000b have the combined sequence content from two different locations (Sargasso Stations 11 and 13) [16]. In this comparison only, GS000a represents the environmental data from Sargasso Station 11 and GS000b that from Sargasso Station 13. Samples where the *in situ *measurement was missing were excluded. This left 55 samples to be compared for temperature and 44 for salinity. The choice of samples for this experiment included no further requirements, because the aim was to demonstrate the accuracy of interpolated data. The interpolations were used as response variables and the *in situ *measurements as explanatory variables. The compared values were expressed in the same units: degrees Celsius for temperature and PSU for salinity. Hence, no further transformation was necessary.

### Protein Domain Searches with Hidden Markov Models

The sequence reads of the GOS metagenome were translated in all six reading frames using the transeq tool from the EMBOSS package [43] with default parameters (version 6.1.0). Hidden Markov Models were selected from the Pfam database (release 24) [35]. Unless stated otherwise, descriptions of HMM models and corresponding TF functions were taken from the Pfam website [44]. Protein domain searches were done with HMMER3 in version 3.0b3 using the default parameters [45]. The results were imported into a relational database. Following the "HMMER3 beta test: User's guide" (Version 3.0b3) [46], *significant *results were defined by the following criteria: 1) domain independent E-value < 0.001, 2) hmm_to-hmm_from > = 20% of model length and 3) the bias should be at least an order of magnitude smaller than the score.

### Single Copy Gene distribution

Samples from GOS were selected to ensure: 1) the filter size used targeted prokaryotes (between 0.1 μm and 0.8 μm) and 2) their origin was not a fresh water environment (based on the habitat type reported in the GOS metadata). Finally, the Sargasso Sea sample GS000a, which is suspected to be contaminated with non-marine *Shewanella *and *Burkholderia *species [47], was removed.

The following samples were excluded from further analysis: GS0 38, 39, 40, 41, 42, 43, 44, 45, 46 and 50. They had extremely low SCG counts, with a maximum per sample average of 1. This was in line with the extremely low number of total sequences in these samples (between 626 and 759 sequences per sample) compared to the rest of the samples (between 11,496 and 692,255 sequences per sample) (Additional file 2, Table S4). A total of 58 samples remained for further analysis (Additional file 2, Table S3). The list of 53 HMMs was based on Ciccarelli et al. 2006 (Additional file 1, Table S3).

### Effect of environment stability on TF content

WOA interpolations were possible for 44 of the 58 GOS samples from the SCG analysis. Additionally, the Mangrove Forest sample GS033 was removed. Environment stability measures are described by the standard deviation of the twelve monthly averages for each interpolated variable at each sampling site (Formula 1 and Additional file 2, Table S5). For GS000b, the average from Sargasso Station 11 and 13 was taken. Stability measures were z-scored (Formula 2) to neutralize the effects of different scales and units [48]. Co-varying stability measures were excluded when their Spearman's rank correlation coefficient (ρ) exceeded 0.6 and the test was statistically significant (p-value < < 0).

The list of TF models was compiled according to Minezaki et al. 2005 [49] (Additional file 1, Table S4). The list contained 40 DNA-Binding Domains (DBDs) and 26 non-DBDs (Additional file 2, Table S6). The models seemed to be rather stable as only one Pfam HMM model had changed since the time of publication in 2005 (PF02573 was merged into or replaced by PF00126). One of the TF HMMs had no significant hits (CtsR, PF05848) and could not be used for the analysis. The raw counts for each TF HMM in each sample (Additional file 2, Table S7) was standardized using the mean of the SCG counts for the respective sample.

$$\sigma =\sqrt{\overset{N}{\underset{i=\text{1}}{\sum }}\frac{{\left(X_{i}-\mu \right)}^{\text{2}}}{N-\text{1}}}$$

Formula 1: Sample standard deviation. The individual values (Xi) are monthly interpolated values for one of the eight environmental parameters. In this study, the standard deviation (σ) was used as a stability measure (the lower the SD, the more stable an environment was considered).

$$z=\frac{x-\mu }{\sigma }$$

Formula 2: Z-score transformation. The raw score (x) is transformed by subtracting the population mean (μ) and dividing by the standard deviation (σ). In this study, each stability measure was treated as a raw score across all samples (the population).

Principal coordinate analysis (PCoA) was used to map the Cartesian distances between the samples back to a 2D plane (Additional file 2, Table S8). The distances between all samples were calculated from their GPS coordinates, using the geographic information system module of the megx.net relational database MegDb [39]. For GS000b, the average of the distance between the two original samples it incorporates and any other sample was taken. PCoA, also known as metric multidimensional scaling, is an ordination method that can map multidimensional data to fewer dimensions to aid interpretation. In this study, the 2D coordinates of each sample (X1, X2) and polynomial terms (up to third-degree terms) thereof represented the spatial components. RDA, which is a multivariate extension of linear regression, was used to calculate the effect of environment stability and space on the total TF content. The standardized TF counts were used as response variables and the four environment stability measures (temperature, salinity, phosphate, silicate), the two spatial coordinates (X1, X2) and their associated polynomial terms (X1^2^, X1^3^, X2^2^, X2^3^) were used as explanatory variables. We applied automatic forward and backward model selection to find the combination of explanatory variables that best explained the variation in the response variables. The combined and independent effect of environment stability and space was tested. The combined model and the independent environmental model both identified temperature stability and phosphate stability as significant explanatory variables. The independent space model identified spatial polynomial terms as significant rather than the X2 from the combined model. We tried to replace X2 in the combined model with combinations of the independent space model; however, no improvement in explained variation or significance levels was observed. Consequently, the combined model was used in further analysis. Variation partitioning was used to separate the effect of environment stability and space. Models and partitions were tested for statistical significance using 1000 permutations of the response data wherever possible. MLR was used to quantify the effect of environment stability and space on individual TFs. The standardized count of each individual TF per sample was used as a response variable. The explanatory variables were the same as for RDA. We compared different model selection methods based on the Akaike information criterion with 1000 steps. Whenever an automatically generated model explained more than 30% of the variation in a TF (R^2 ^> 0.3), we tried to manually improve it by removing explanatory variables with low significance (p-value > 0.1).

## List of Abbreviations

DBD: DNA-binding domain; GOS: Global Ocean Sampling; MLR: Multiple linear regression; RDA: Redundancy analysis; SCG: Single-copy gene; TF: Transcription factor; WOA: World Ocean Atlas.

## Competing interests

The authors declare that they have no competing interests.

## Authors' contributions

IK and RK conceived and planned the study. IK performed the analysis and drafted the manuscript. AR participated in statistical analysis. JW participated in the Hidden Markov Model searches. PLB participated in the formulation of the study and helped draft the manuscript. FOG coordinated the work and finalized the manuscript. All authors read and approved the final manuscript.

## Authors' information

IK is a bioinformatician focusing on functional metagenomics and data integration. RK is a bioinformatician focusing on data integration and standardization. AR is a microbial ecologist with strong background in multivariate statistics. JW is an informatician with strong background in bioinformatic pipeline development. PLB applies a background in biochemistry, cell biology and marine microbiology to the study of ecological genomics. FOG is a Professor of Bioinformatics at Jacobs University and group leader of the Microbial Genomics and Bioinformatics Group at the Max Planck Institute for Marine Microbiology. FOG and RK are active members of the Genomics Standards Consortium.

## Supplementary Material

Additional file 1 **A PDF file containing figures and tables that further describe and visualize the analysis in more detail**. **Figures: **Figure S1: Distribution of SCGs against the number of sequences per sample. Figure S2: Coefficient of variation of SCGs against the number of sequences per sample. Figure S3: Seven descriptive statistic functions of SCG counts against the number of sequences per sample. Figure S4: Correlation of environmental stability variables to each other. **Tables: **Table S1: A list of SCG models that were identified as outliers. Table S2: Correlation coefficients of environmental stability variables Table S3: A list of SCG HMMs based on Ciccarelli et al. (2006). Table S4: TF models after Minezaki et al. (2005). Click here for file

Additional file 2 **A zip file containing data and R code for reproducing the analysis in this study. Contents are listed below: Rcode.txt - R code used for the analysis in this publication**. Table_S1.csv - Interpolated and measured values for temperature and salinity. Table_S2.csv - Monthly interpolations for GS041. Table_S3.csv - SCG raw counts. Table_S4.csv -Number of sequences per sample. Table_S5.csv - Environmental stability measures. Table_S6.csv - TF model categories (DBD. non-DBD). Table_S7.csv - TF raw counts. Table_S8.csv - Cartesian distance between GOS samples. Click here for file
