# Supplementary material for: Quantifying the effect of environment stability on the transcription factor repertoire of marine microbes
Source: Microb Inform Exp. 2011 Sep 7;1:9. doi: 10.1186/2042-5783-1-9 (PMC3372289; doi:10.1186/2042-5783-1-9)
Supplement: Additional file 1 — A PDF file containing figures and tables that further describe and visualize the analysis in more detail. Figures: Figure S1: Distribution of SCGs against the number of sequences per sample. Figure S2: Coefficient of variation of SCGs against the number of sequences per sample. Figure S3: Seven descriptive statistic functions of SCG counts against the number of sequences per sample. Figure S4: Correlation of environmental stability variables to each other. Tables: Table S1: A list of SCG models that were identified as outliers. Table S2: Correlation coefficients of environmental stability variables Table S3: A list of SCG HMMs based on Ciccarelli et al. (2006). Table S4: TF models after Minezaki et al. (2005). [file 2042-5783-1-9-S1.DOC]

### List of Figures:

Figure S1: Distribution of SCGs against the number of sequences per sample.

Figure S2: Coefficient of variation of SCGs against the number of sequences per sample.

Figure S3: Seven descriptive statistic functions of SCG counts against the number of sequences per sample.

Figure S4: Correlation of environmental stability variables to each other.

### List of Tables:

Table S1: A list of SCG models that were identified as outliers.

Table S2: Correlation coefficients of environmental stability variables

Table S3: A list of SCG HMMs based on Ciccarelli et al. (2006).

Table S4: TF models after Minezaki et al. (2005).


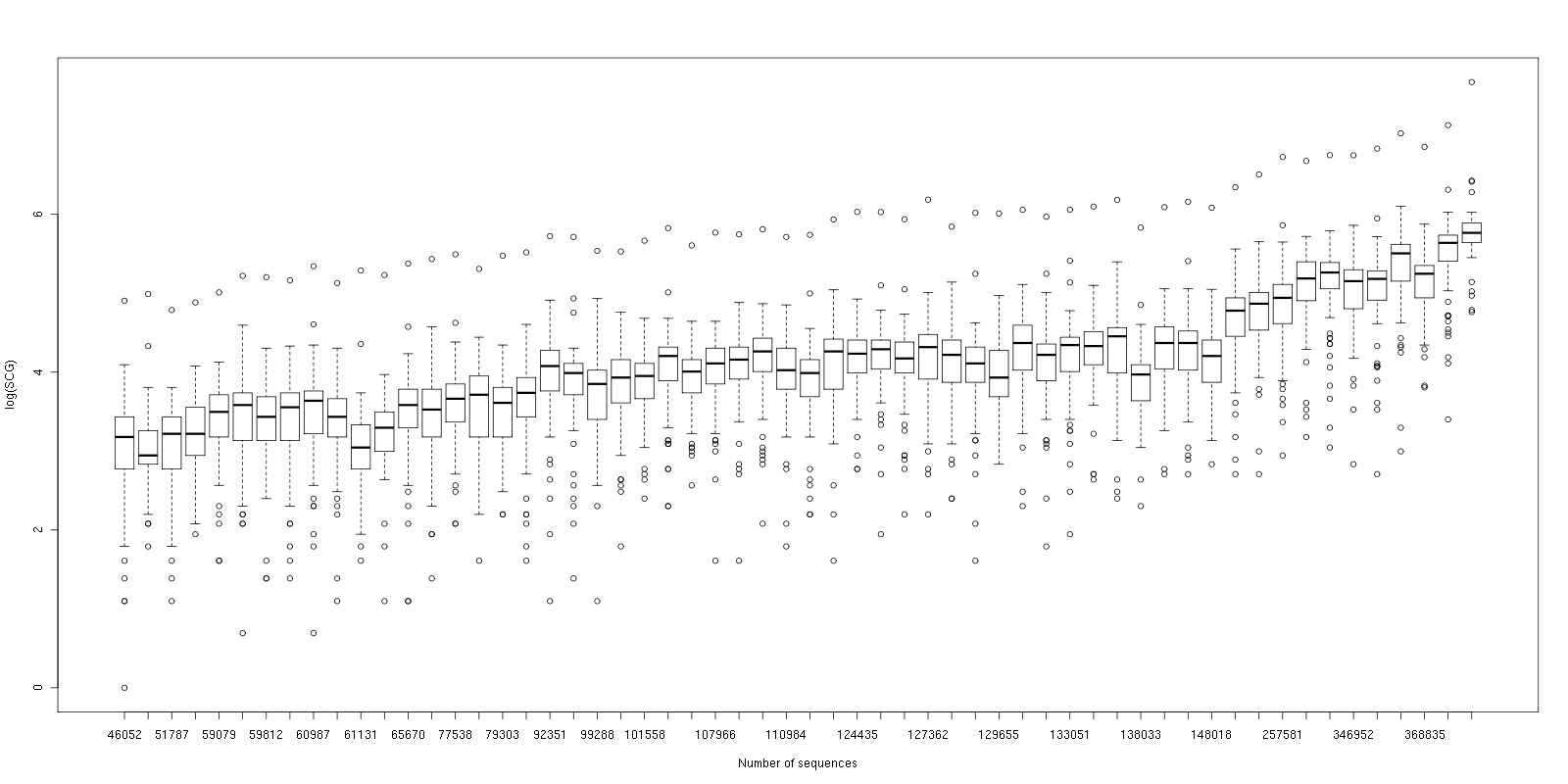
Figure S1: Distribution of SCGs against the number of sequences per sample.

The absolute counts of SCGs per sample were log-transformed (Y axis). In the boxplots, the whiskers’ ends correspond roughly to ±2 standard deviations around the mean. More concretely, they denote the furthest data points still within 1.5 times the interquartile range ([IQR](http://en.wikipedia.org/wiki/Interquartile_range)) of the first (Q1) and the third quartile (Q3). The IQR is calculated as follows: IQR = Q3-Q1. The dots represent SCGs that lie outside these ranges and are therefore considered outliers. A list of the outliers is available in Additional file 1, Table S1.

Table S1: A list of SCG HMM models that were identified as outliers.

The number of samples in which the model was an outlier and the percentage of all samples (58 in total) are presented.

| **SCG model** | **number of samples** | **percent of all samples** |
| --- | --- | --- |
| **Above 1.5 IQR of Q3** | |  |
| Usg | 1 | 2 |
| if_n2 | 4 | 7 |
| Reca | 18 | 31 |
| ruvb_n | 58 | 98 |
| **Below 1.5 IQR of Q1** | |  |
| Rimm | 1 | 2 |
| Secg | 4 | 7 |
| Ruvc | 6 | 10 |
| duf150 | 7 | 12 |
| trigger_c | 9 | 15 |
| exonuc_vii_s | 15 | 25 |
| tyr_deacylase | 20 | 34 |
| glutr_n | 31 | 53 |
| duf177 | 35 | 59 |
| Hrca | 41 | 69 |
| glutr_dimer | 46 | 78 |
| ribosomal_s20p | 55 | 93 |


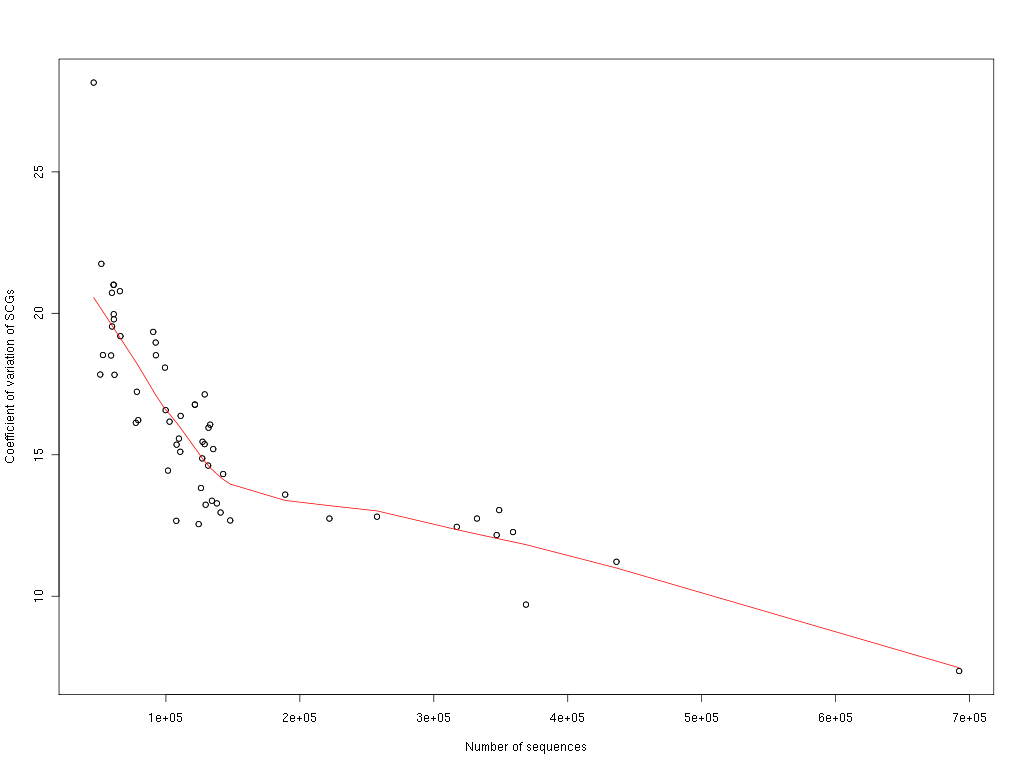
Figure S2: Coefficient of variation of SCGs against the number of sequences per sample.

The variation within SCG numbers decreases with increasing number of sequences, supporting the idea that deeper sequencing delivers more stable data.
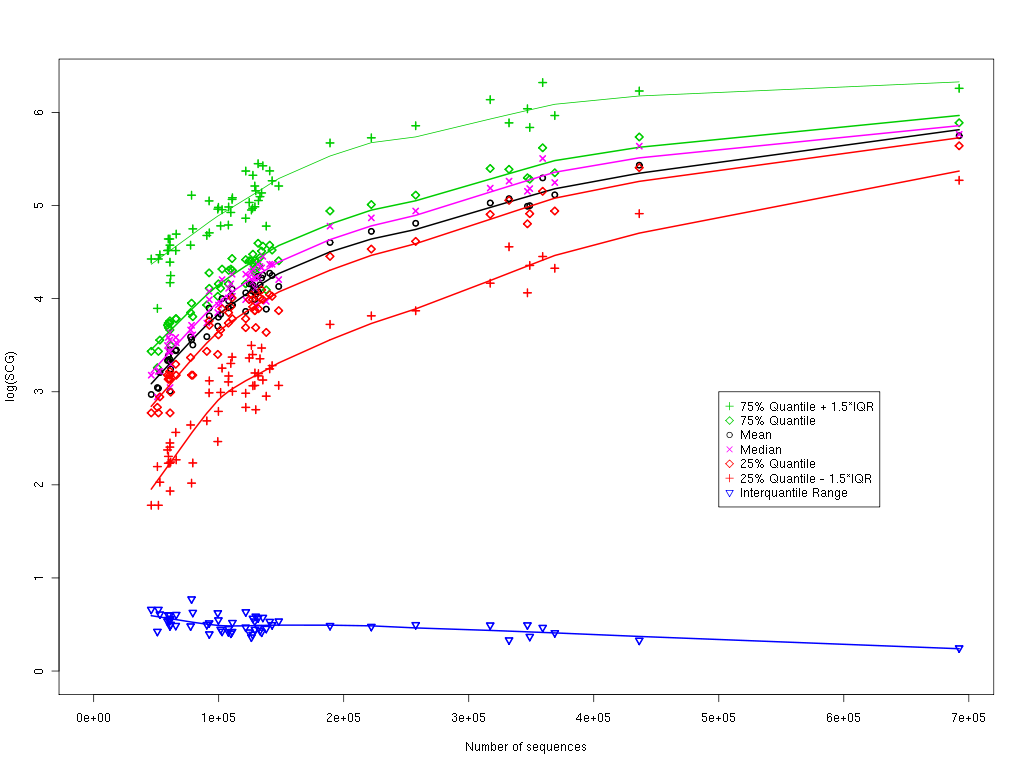
Figure S3: Seven descriptive statistic functions of SCG counts against the number of sequences per sample.

The absolute counts of SCGs per sample were log-transformed (Y axis).


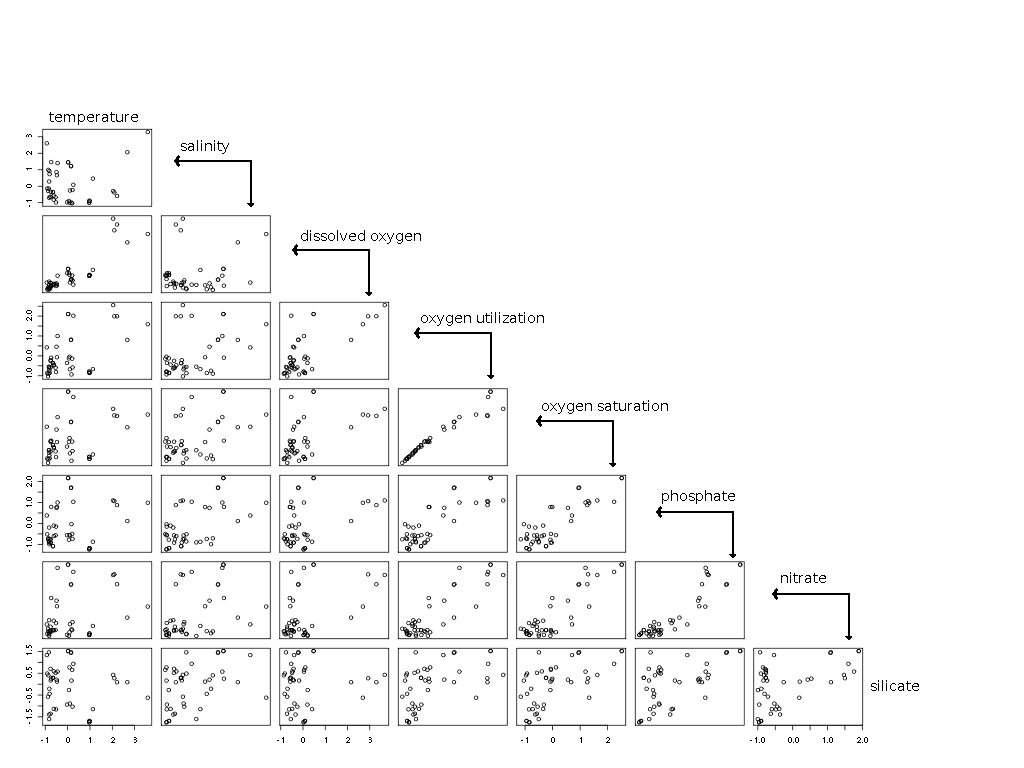


Figure S4 Correlation of environmental stability variables.

This is a visual representation - a roughly diagonal line in any direction would mean a considerable correlation.

Table S2 Correlation coefficients of environmental stability variables.

Variable pairs with Spearman correlation coefficient above 0.6 are shown.

| **stability measures** | | **rho** | **p-value** |
| --- | --- | --- | --- |
| temperature | oxygen_dissolved | 0.81 | 3.27E-011 |
| Oxygen_utilization | oxygen_saturation | 0.99 | 2.20E-016 |
| Oxygen_utilization | phosphate | 0.70 | 1.31E-007 |
| Oxygen_saturation | phosphate | 0.70 | 1.31E-007 |
| Oxygen_utilization | nitrate | 0.66 | 9.51E-007 |
| Oxygen_saturation | nitrate | 0.66 | 8.97E-007 |
| phosphate | nitrate | 0.85 | 2.96E-013 |

Table S3: A list of SCG HMMs based on Ciccarelli et al. (2006).

| **Accession** | **Pfam Id** | **Model length** | **Average domain length** |
| --- | --- | --- | --- |
| PF00189 | Ribosomal_S3_C | 85 | 82.0 |
| PF00252 | Ribosomal_L16 | 133 | 113.7 |
| PF00417 | Ribosomal_S3_N | 66 | 63.3 |
| PF00453 | Ribosomal_L20 | 108 | 101.7 |
| PF00475 | IGPD | 145 | 144.3 |
| PF00542 | Ribosomal_L12 | 68 | 67.0 |
| PF00584 | SecE | 57 | 56.6 |
| PF00745 | GlutR_dimer | 101 | 100.6 |
| PF00825 | Ribonuclease_P | 111 | 109.0 |
| PF00829 | Ribosomal_L21p | 96 | 95.0 |
| PF00831 | Ribosomal_L29 | 58 | 57.4 |
| PF00886 | Ribosomal_S16 | 62 | 57.7 |
| PF00889 | EF_TS | 221 | 180.3 |
| PF01016 | Ribosomal_L27 | 81 | 80.7 |
| PF01192 | RNA_pol_Rpb6 | 57 | 54.4 |
| PF01196 | Ribosomal_L17 | 97 | 100.8 |
| PF01245 | Ribosomal_L19 | 113 | 113.0 |
| PF01250 | Ribosomal_S6 | 92 | 91.7 |
| PF01281 | Ribosomal_L9_N | 48 | 47.9 |
| PF00828 | Ribosomal_L18e | 129 | 118.9 |
| PF01628 | HrcA | 224 | 219.4 |
| PF01649 | Ribosomal_S20p | 84 | 82.0 |
| PF01668 | SmpB | 68 | 67.2 |
| PF01746 | tRNA_m1G_MT | 186 | 190.6 |
| PF01765 | RRF | 165 | 163.1 |
| PF01782 | RimM | 84 | 83.7 |
| PF02033 | RBFA | 104 | 104.9 |
| PF02075 | RuvC | 149 | 147.7 |
| PF02092 | tRNA_synt_2f | 549 | 541.7 |
| PF02130 | UPF0054 | 145 | 142.1 |
| PF02132 | RecR | 41 | 41.0 |
| PF02357 | NusG | 92 | 98.2 |
| PF02410 | DUF143 | 100 | 98.5 |
| PF02542 | YgbB | 157 | 156.4 |
| PF02565 | RecO_C | 118 | 151.4 |
| PF02576 | DUF150 | 141 | 138.8 |
| PF02580 | Tyr_Deacylase | 145 | 142.8 |
| PF02609 | Exonuc_VII_S | 53 | 52.9 |
| PF02620 | DUF177 | 119 | 114.4 |
| PF02686 | Glu-tRNAGln | 72 | 72.4 |
| PF02912 | Phe_tRNA-synt_N | 73 | 72.6 |
| PF02978 | SRP_SPB | 104 | 100.3 |
| PF03147 | FDX-ACB | 94 | 94.2 |
| PF03483 | B3_4 | 174 | 167.7 |
| PF03484 | B5 | 70 | 70.0 |
| PF03726 | PNPase | 83 | 81.9 |
| PF03840 | SecG | 74 | 73.3 |
| PF03948 | Ribosomal_L9_C | 87 | 86.9 |
| PF04760 | IF2_N | 54 | 52.0 |
| PF05201 | GlutR_N | 152 | 148.4 |
| PF05496 | RuvB_N | 234 | 212.7 |
| PF05698 | Trigger_C | 162 | 154.7 |
| PF00154 | RecA | 323 | 233.9 |

Table S4: A list of TF HMMs based on Minezaki et al. (2005).

| **Accession** | **Pfam Id** |
| --- | --- |
| PF00027 | cNMP_binding |
| PF00072 | Response_reg |
| PF00126 | HTH_1 |
| PF00155 | Aminotran_1_2 |
| PF00158 | Sigma54_activat |
| PF00165 | HTH_AraC |
| PF00171 | Aldedh |
| PF00196 | GerE |
| PF00325 | Crp |
| PF00356 | LacI |
| PF00376 | MerR |
| PF00392 | GntR |
| PF00440 | TetR_N |
| PF00480 | ROK |
| PF00486 | Trans_reg_C |
| PF00532 | Peripla_BP_1 |
| PF00717 | Peptidase_S24 |
| PF01022 | HTH_5 |
| PF01047 | MarR |
| PF01316 | Arg_repressor |
| PF01325 | Fe_dep_repress |
| PF01340 | MetJ |
| PF01371 | Trp_repressor |
| PF01380 | SIS |
| PF01381 | HTH_3 |
| PF01402 | RHH_1 |
| PF01418 | HTH_6 |
| PF01475 | FUR |
| PF01590 | GAF |
| PF01619 | Pro_dh |
| PF01722 | BolA |
| PF01726 | LexA_DNA_bind |
| PF01965 | DJ-1_PfpI |
| PF01978 | TrmB |
| PF02082 | Rrf2 |
| PF02237 | BPL_C |
| PF02311 | AraC_binding |
| PF02742 | Fe_dep_repr_C |
| PF02805 | Ada_Zn_binding |
| PF02863 | Arg_repressor_C |
| PF02954 | HTH_8 |
| PF03099 | BPL_LplA_LipB |
| PF03459 | TOBE |
| PF03466 | LysR_substrate |
| PF03472 | Autoind_bind |
| PF03551 | PadR |
| PF03704 | BTAD |
| PF03749 | SfsA |
| PF03965 | Pencillinase_R |
| PF04023 | FeoA |
| PF04198 | Sugar-bind |
| PF04299 | FMN_bind_2 |
| PF04397 | LytTR |
| PF04967 | HTH_10 |
| PF05068 | MtlR |
| PF05247 | FlhD |
| PF05443 | ROS_MUCR |
| PF05848 | CtsR |
| PF06018 | CodY |
| PF06338 | ComK |
| PF06506 | PrpR_N |
| PF06923 | GutM |
| PF06956 | RtcR |
| PF06988 | NifT |
| PF07417 | Crl |
